# Topoisomerase inhibitors suppressed lithocholic acid-induced promotion of transformation in BALB/3T3.

**DOI:** 10.1038/bjc.1987.251

**Published:** 1987-11

**Authors:** M. Kaneko, J. Horikoshi

**Affiliations:** Biophysics Division, National Cancer Center Research Institute, Tokyo, Japan.


Br. J. Cancer (1987), 56, 614–616 © The Macmillan Press Ltd., 1987

SHORT COMMUNICATION

### Topoisomerase inhibitors suppressed lithocholic acid-induced promotion of transformation in BALB/3T3

M. Kaneko & J. Horikoshi

*Biophysics Division, National Cancer Center Research Institute, Tsukiji 5-1-1, Chuo-ku, Tokyo, Japan.*

Epidemiological studies have suggested that a high fat diet and the metabolic activity of gut flora are major factors contributing to development of colon cancer (Reddy, 1981). Studies in rats have showed that bile acids such as deoxycholic acid and lithocholic acid enhance tumour formation in the colon (Reddy, 1981), stomach (Kobori *et al.*, 1984) and liver (Cameron *et al.*, 1981). Moreover, bile acids have been found to have initiating (Kelsey *et al.*, 1979) or enhancing (Kaibara *et al.*, 1984) effects in transformation of cultured cells. We have been attempting to clarify the mechanism of these biological effects by studying DNA lesions in cultured cells exposed to bile acids. Previously, we found that various bile acids induced DNA single strand breaks, the extent of which depended on their degree of incorporation into cultured cells, and that most of the single strand breaks (up to 80%) were associated with protein–DNA cross linking. We also found that the induction of single strand breaks was effectively suppressed by topoisomerase inhibitors such as novobiocin (Nov), nalidixic acid (Nal), oxolinic acid (Oxl) and coumermycin A (Kaneko *et al.*, 1986). To examine whether these inhibitors also inhibited cell transformation, we investigated their effects on promotion of transformation by lithocholic acid (LC) in Balb/3T3 cells. Here we show that Nal and Oxl efficiently reduced the promotion of transformation by LC initiated by 3-methylcholanthrene (MC) in BALB/3T3 fibroblasts.

The BALB/3T3, A31-1-1 *in vitro* transformation system was used. This system has been well characterized (Kakunga, 1973) and used as a model system in two-step carcinogenesis experiments (Kennedy & Little, 1978). The cells were grown in Eagle's minimum essential medium supplemented with 10% heat-inactivated foetal calf serum. For transformation, inocula of $1 \times 10^4$ cells of the A31-1-1 BALB/3T3 cell line (passage 3 after receipt as a gift from Dr T. Kuroki) were seeded into ten 60 mm cell culture dishes per experimental group. After 24 h, the cultures were treated with MC (Spectrum Chem, MFG. Corp., Redondo Beach, Ca.) for 72 h at 37°C. Then the medium was changed, and culture was continued for 4 weeks in the presence or absence of LC (Sigma Chem. Co., St Louis, Mo.) with or without Oxl or Nal (Sigma Chem. Co.) as indicated. The medium was renewed twice a week. Type III foci (Reznikoff *et al.*, 1973) in each plate were scored as transformants with the aid of a dissecting microscope. The criteria adopted for them are that cells are highly condensed, heavily piled upon one another, and randomly oriented. In addition, areas of patchy, dense growth without criss-crossing were often observed but they were clearly distinct from the above foci and were not scored. MC and LC were dissolved in dimethylsulfoxide (Pierce, Rockfield, Il.). Nal and Nov were dissolved in distilled water and Oxl was dissolved in 40 mM NaOH. The promoter and inhibitors were present throughout the expression period after the treatment with MC. Four dishes per group seeded with 200 cells were treated in parallel to assess the effects of the various treatments upon colony forming efficiency. Colonies in test dishes were scored after incubation for one week.

Five experiments were carried out to determine the effects of Nal and Oxl on MC-induced transformation and LC-induced promotion of transformation, together with confirmation of LC-induced promotion (Kaibara *et al.*, 1984) and three of these experiments (A, B and C) are shown in Table I. As shown in expts. A, B and C, Oxl and Nal clearly suppressed transformation promoted by LC. Surprisingly, the suppression was frequently to below the level of transformation induced by MC only. In most experiments, these inhibitors also suppressed transformation induced by MC only. On the other hand, novobiocin, which also suppressed induction of DNA strand breaks by LC, little affected LC-induced promotion of transformation when added at 75 $\mu$M, which was the highest concentration possible because of its high toxicity (Table II). LC alone (40 $\mu$M) induced transformation on prolonged treatment (expts. A, B and C), but not on a single treatment (data not shown). Therefore, it may have weak initiating activity in BALB/3T3 cells, as it does in hamster embryo cells (Kelsey *et al.*, 1979). The transformation induced by prolonged treatment with LC was also suppressed by Nal (expt. C) or Oxl (expt. B). Thus, the prokaryotic topoisomerase inhibitors Nal and Oxl, but not Nov, suppressed transformation induced by MC or LC; and also promotion by LC after initiation by MC in BALB/3T3 cells as well as suppressing LC-induced DNA strand breaks in cultured human fibroblasts (Kaneko *et al.*, 1986).

Neither the way in which LC acts on inducing DNA strand breaks and transformation nor the way in which Oxl and Nal act on suppressing them are known. The following observations suggest that topoisomerase participate in these processes: First, Oxl, Nal and Nov have been reported to inhibit 4′-(9-acridinylamino)-methanesulfon-m-anisidide (m-AMSA) induced DNA-protein cross linking mediated by topoisomerase II (Pommier *et al.*, 1984, Nelson *et al.*, 1984). Moreover, we found previously that they also inhibited DNA-protein cross linking induced by LC in cultured fibroblasts (Kaneko *et al.*, 1986). Second, in a preliminary experiment we found that in a cell free system LC inhibited topoisomerase assayed by measuring relaxation of closed circular DNA (unpublished data). Nal may inhibit LC-induced processes by reducing the level of ATP, since it was found to interfere with oxidative phosphorylation in isolated rat liver mitochondria and to inhibit ATP synthesis (Gallagher *et al.*, 1986). However, its effect may not be mediated by decrease in the ATP level, because DNA strand breaks in cultured fibroblasts were increased 2- to 3-fold in the presence of an uncoupler such as oligomycin or dinitrophenol (unpublished results). In addition, it was shown that Nal and Oxl inhibit topoisomerase II in a cell free system from HeLa cells at high concentration (Miller *et al.*, 1981) but that Nal does not inhibit topoisomerase from *D. melanogaster* (Hsie & Brutlag, 1980). Based on these

Correspondence: M. Kaneko.
Received 30 March 1987; and in revised form, 5 June 1987.

**Table I** Effects of oxolinic acid and nalidixic acid on lithocholic acid-induced promotion of transformation initiated by 3-methylcholanthrene

| Expt. | Treatment: Initiation | Treatment: Promotion[a] | Plating efficiency[b] | No. of type III foci/dish[b] | Fractions of dishes without foci | Frequency of transformation |
|---|---|---|---|---|---|---|
| | | | /200 | | /10 | $\times 10^{-4}$ |
| A. | None | None | 104.0± 8.8 | 0.0 | 10 | 0.0 |
| | | LC | 96.5± 9.2 | 0.4±1.0 | 8 | 0.86± 2.10 |
| | | Oxl | 110.3± 9.5 | 0.0 | 10 | 0.0 |
| | | LC+Oxl | 98.2± 6.8 | 0.4±0.5 | 6 | 0.84± 1.10 |
| | MC(1 μg ml$^{-1}$) | None | 76.8± 3.0 | 0.8±0.8 | 4 | 2.16± 2.14 |
| | | LC | 76.0±13.1 | 7.3±1.8 | 0 | 19.98± 4.92[c] |
| | | Oxl | 76.5± 7.3 | 0.3±0.5 | 7 | 0.82± 1.30 |
| | | LC+Oxl | 68.7± 7.4 | 0.8±0.7 | 3 | 2.36± 2.02[d] |
| B. | None | None | 113.5± 8.9 | 0.0 | 10 | 0.0 |
| | | LC | 107.8± 6.8 | 0.6±0.8 | 6 | 1.1 ± 1.6 |
| | | LC+Oxl | 110.3± 8.1 | 0.1±0.3 | 9 | 0.2 ± 0.6 |
| | MC(1 μg ml$^{-1}$) | None | 62.8± 8.5 | 2.6±2.0 | 4 | 8.3 ± 6.4 |
| | | LC | 65.8± 3.5 | 5.9±3.3 | 0 | 17.9 ±10.0 |
| | | Oxl | 70.0± 5.4 | 0.7±0.8 | 5 | 2.0 ± 2.3[e] |
| | | LC+Oxl | 69.0± 1.6 | 1.7±1.4 | 3 | 4.9 ± 4.1[d] |
| C. | None | None | 77.0± 5.9 | 0.0 | 10 | 0.0 |
| | | LC | 69.8± 3.3 | 1.5±1.2 | 2 | 4.3 ± 3.4 |
| | | Nal | 81.0± 5.0 | 0.0 | 10 | 0.0 |
| | | LC+Nal | 68.3± 9.3 | 0.1±0.3 | 9 | 0.3 ± 0.9 |
| | MC(1 μg ml$^{-1}$) | None | 28.8± 5.0 | 0.9±2.7 | 9 | 6.3 ±18.9 |
| | | LC | 24.8± 3.0 | 8.1±2.6 | 0 | 65.3 ±21.0[c] |
| | | LC+Nal | 51.0±10.0 | 0.3±0.6 | 8 | 1.2 ± 2.4[d] |
| | MC(5 μg ml$^{-1}$) | None | 30.0± 8.1 | 2.9±1.8 | 0 | 19.3 ±12.0 |
| | | LC | 27.0± 1.9 | 18.2±6.7 | 0 | 134.8 ±49.6[f] |
| | | Nal | 19.8± 3.7 | 0.6±0.9 | 9 | 6.1 ± 9.2[g] |
| | | LC+Nal | 45.8± 7.0 | 0.1±0.3 | 6 | 0.4 ± 1.2[h] |

[a]Concentrations used were as follows: LC, 40 μM; Oxl, 100 μM; Nal, 0.5 mM; [b]Values are means±s.d. for 4 dishes (plating efficiency) or 10 dishes (transformation); [c]Differs from MC(1 μg ml$^{-1}$) ($P<0.001$ for expt. A, $P<0.001$ for expt. C) by $t$-test; [d]Differs from MC(1 μg ml$^{-1}$) plus LC ($P<0.001$ for both expt. A and C and $P<0.01$ for expt. B); [e]Differs from MC(1 μg ml$^{-1}$) ($P<0.01$); [f]Differs from MS(5 μg ml$^{-1}$) ($P<0.001$); [g]Differs from MC(5 μg ml$^{-1}$) ($P<0.02$); [h]Differs from MC(5 μg ml$^{-1}$) plus LC ($P<0.001$).

**Table II** Effects of novobiocin on lithocholic acid-induced promotion of transformation initiated by 3-methylcholanthrene

| Treatment: Initiation | Treatment: Promotion[a] | Plating efficiency[b] | No. of type III foci/dish[b] | Fractions of dishes without foci | Frequency of transformation |
|---|---|---|---|---|---|
| | | /200 | | /10 | $\times 10^{-4}$ |
| None | None | 107.5± 9.4 | 0.0 | 10 | 0.0 |
| | LC | 101.3± 5.4 | 2.7±1.7 | 0 | 5.3± 3.4 |
| | Nov | 97.8± 2.5 | 0.0 | 10 | 0.0 |
| | LC+Nov | 100.8±10.0 | 4.8±7.5 | 0 | 9.5±14.9 |
| MC(1 μg ml$^{-1}$) | None | 73.5± 6.9 | 1.0±1.3 | 5 | 2.7± 3.5 |
| | LC | 74.5± 4.3 | 20.1±3.9 | 0 | 54.0±10.5 |
| | Nov | 71.3±10.2 | 4.4±2.3 | 1 | 12.3±28.6 |
| | LC+Nov | 62.3± 4.5 | 17.2±6.7 | 0 | 55.2±21.5 |

[a]Concentration used were: LC, 40 μM; Nov, 75 μM; [b]As foot-note to **Table I**.

findings we propose that LC, Oxl and Nal may act in the following way on the induction of DNA strand breaks and transformation and on their suppression: First, LC might interfere with some function of topoisomerase and this interference might induce DNA-protein cross linking. Second, LC-induced DNA-protein cross linking might play a role in LC-induced promotion of transformation. Third, the interaction between LC and topoisomerases might be inhibited directly or indirectly by Oxl or Nal, or cells treated with Oxl or Nal might be able to repair LC-induced damage. Experiments using a cell-free system are necessary to investigate these possibilities and will help in determining whether topoisomerases actually participate in transformation induced by MC alone.

Recently, there has been increasing interest in the function ot topoisomerases. There are suggestions (Wang, 1985) that topoisomerase I is essential for RNA transcription and that topoisomerase II is essential to DNA replication. Also, it is reported that topoisomerase II is a major constituent of nuclear scaffold (Earnshaw *et al.*, 1985) and is essential to mitosis (Holm *et al.*, 1985). Modification of topoisomerase II by an antineoplastic agent such as m-AMSA was reported to induce preferential cleavage at a DNase I hypersensitive region (~270 bp upstream of Ori) of SV40 chromatin in monkey kidney cells (Yang *et al.*, 1985) and sister chromatid exchange in Chinese hamster cells (Dillehay *et al.*, 1987). On the other hand, recently, the effect of 4$\beta$-phorbol 12,13-dibutyrate on differentiation of HL-60 cells was found to be suppressed by inhibitors of topoisomerase II such as Nov and m-AMSA and activation of topoisomerase II due to phosphorylation by protein kinase C was suggested to be involved in the differentiation process (Sahyoun *et al.*, 1986). Thus, topoisomerases are considered to be important not only in differentiation but also in carcinogenesis after activation or inactivation. But there have been no previous suggestions that topoisomerases participate in promotion of carcinogenesis or in the whole process of carcinogenesis. Therefore, it is important to examine whether they are essential for induction of transformation by MC and induction of promotion by LC.

We are grateful to Dr T. Kuroki, University of Tokyo, Medical Inst., for advice in the assay of cell transformation and to Drs M. Kodama and T. Kimura, of this division, for valuable discussions.

### References

CAMERON, R., IMAIDA, K. & ITO, N. (1981). Promotive effects of deoxycholic acid on hepatocarcinogenesis initiated by diethylnitrosoamine in male rats. *Gann*, **72**, 635.

DILLEHAY, L.E., DENSTMAN, S.T. & WILLIAMS, J.R. (1987). Cell cycle dependence of sister chromatid exchange induction by DNA topoisomerase II inhibitors in Chinese hamster V79 cells. *Cancer Res.*, **47**, 206.

EARNSHAW, W.C., HALLIGAN, B., COOKE, C.A., HECK, M.M.S. & LIU, L.F. (1985). Topoisomerase II is a structural component of mitotic chromosome scaffolds. *J. Cell Biol.*, **100**, 1706.

GALLAGHER, M., WEINBERG, R. & SIMPSON, M.V. (1986). Effect of the bacterial DNA gyrase inhibitors, novobiocin, nalidixic acid, and oxolinic acid on oxidative phosphorylation. *J. Biol. Chem.*, **261**, 8604.

HOLM, C., GOTO, T., WANG, J.C. & BOTSTEIN, D. (1985). DNA topoisomerase II is required at the time of mitosis in yeast. *Cell*, **41**, 553.

HSIE, T.S. & BRUTLAG, D. (1980). ATP dependent DNA topoisomerase from *D. melanogaster* reversibly catenates duplex DNA rings. *Cell*, **21**, 115.

KAIBARA, N., YURUGI, E. & KOGA, S. (1984). Promoting effect of bile acids on the chemical transformation of C3H/10T/1/2 fibroblasts *in vitro*. *Cancer Res.*, **44**, 5482.

KAKUNAGA, T. (1973). A quantitative system for assay of malignant transformation by chemical carcinogens using a clone derived from Balb/3T3. *Int. J. Cancer*, **12**, 463.

KANEKO, M., HORIKOSHI, J. & KODAMA, M. (1986). Topoisomerase inhibitors reduced DNA single strand breaks induced by bile acids in cultured human fibroblasts. *Cancer J.*, **1**, 169.

KELSEY, M.I. & PIENTA, R.J. (1979). Transformation of hamster embryo cells by cholesterol-epoxide and lithocholic acid. *Cancer Lett.*, **6**, 143.

KENNEDY, A.R. & LITTLE, J.B. (1978). Protease inhibitors suppress radiation-induced malignant transformation *in vitro*. *Nature*, **276**, 825.

KOBORI, O., SHIMIZU, T., MAEDA, M. & 4 others (1984). Enhancing effect of bile and bile acid on stomach tumorigenesis induced by N-methyl-N′-nitro-N-nitrosoguanidine in Wistar rats. *J. Natl Cancer Inst.*, **73**, 853.

MILLER, K.G., LIU, L.F. & ENGLUND, P.T. (1981). A homogeneous type II DNA topoisomerase from Hela cell nuclei. *J. Biol. Chem.*, **256**, 9334.

NELSON, E.M., TWEY, K.M. & LIU, L.F. (1984). Mechanism of antitumor drug action; Poisoning of mammalian DNA topoisomerase II on DNA by 4′-(9-acridinylamino)-methansulfon-m-anisidide. *Proc. Natl Acad. Sci. USA.*, **81**, 1361.

POMMIER, Y., SCHWARTZ, R.E., KOHN, K.W. & ZWELLING, L.A. (1984). Formation and rejoining of deoxyribonucleic acid double-strand breaks induced in isolated nuclei by antineoplastic intercalating agents. *Biochemistry*, **23**, 3194.

REDDY, B.S. (1981). Dietary fat and its relationship to large bowel cancer. *Cancer Res.*, **41**, 3700.

REZNIKOFF, C.A., BERTRAM, J.S., BRANKOW, D.W. & HEIDERBERGER, C. (1973). Quantitative and qualitative studies of chemical tranformation of cloned C3H mouse embryo cells sensitive to postconfluence inhibition of cell division. *Cancer Res.*, **33**, 3239.

SAHYOUN, N., WOLF, M., BESTERMAN, J. & 5 others (1986). Protein kinase C phosphorylates topoisomerase II: Topoisomerase activation and its possible role in phorbol ester-induced differentiation of HL-60 cells. *Proc. Natl Acad. Sci. USA.*, **83**, 1603.

WANG, J.C. (1985). DNA topoisomerase. *Ann. Rev. Biochem.*, **54**, 665.

YANG, L., ROWE, T., NELSON, E.M. & LIU, L.F. (1985). *In vivo* mapping of DNA topoisomerase II-specific cleavage sites on SV40 chromatine. *Cell*, **41**, 127.


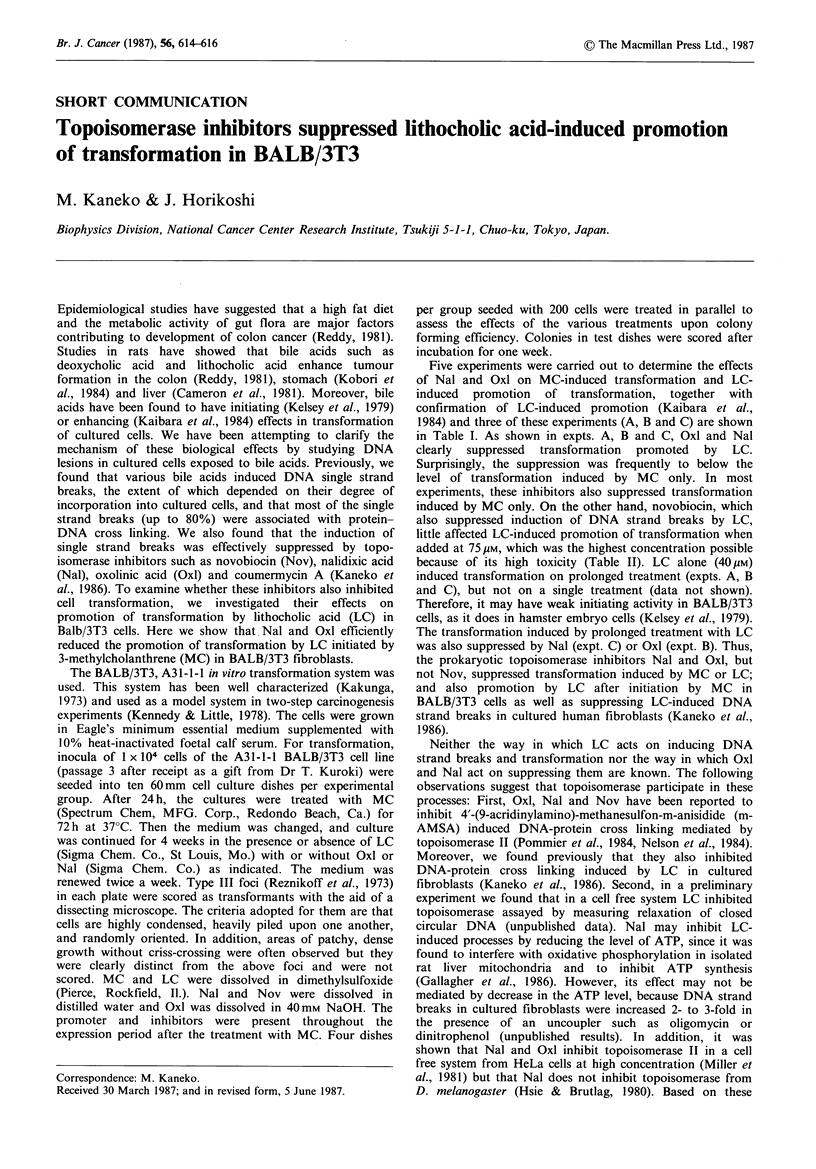

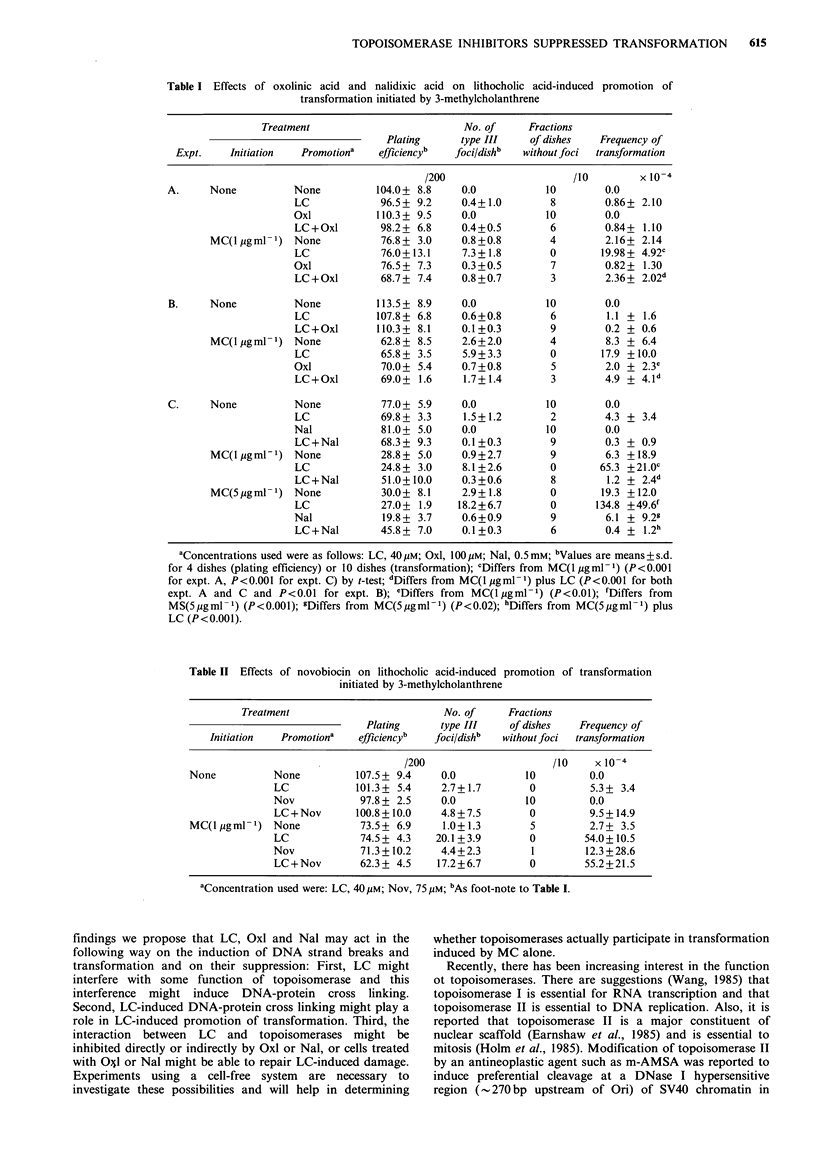

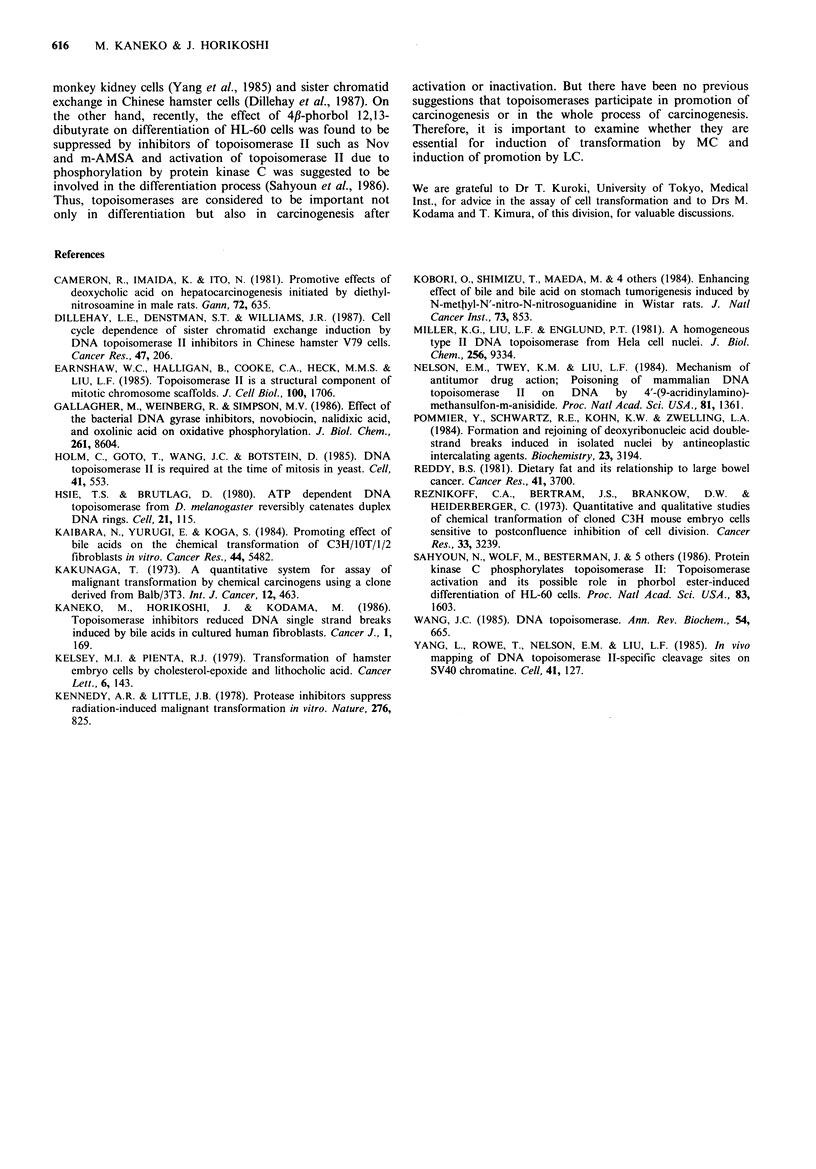


## References

[OCR_00269] Cameron R., Imaida K., Ito N. (1981). Promotive effects of deoxycholic acid on hepatocarcinogenesis initiated by diethylnitrosamine in male rats.. Gan.

[OCR_00274] Dillehay L. E., Denstman S. C., Williams J. R. (1987). Cell cycle dependence of sister chromatid exchange induction by DNA topoisomerase II inhibitors in Chinese hamster V79 cells.. Cancer Res.

[OCR_00280] Earnshaw W. C., Halligan B., Cooke C. A., Heck M. M., Liu L. F. (1985). Topoisomerase II is a structural component of mitotic chromosome scaffolds.. J Cell Biol.

[OCR_00285] Gallagher M., Weinberg R., Simpson M. V. (1986). Effect of the bacterial DNA gyrase inhibitors, novobiocin, nalidixic acid, and oxolinic acid, on oxidative phosphorylation.. J Biol Chem.

[OCR_00291] Holm C., Goto T., Wang J. C., Botstein D. (1985). DNA topoisomerase II is required at the time of mitosis in yeast.. Cell.

[OCR_00296] Hsieh T., Brutlag D. (1980). ATP-dependent DNA topoisonmerase from D. melanogaster reversibly catenates duplex DNA rings.. Cell.

[OCR_00301] Kaibara N., Yurugi E., Koga S. (1984). Promoting effect of bile acids on the chemical transformation of C3H/10T1/2 fibroblasts in vitro.. Cancer Res.

[OCR_00306] Kakunaga T. (1973). A quantitative system for assay of malignant transformation by chemical carcinogens using a clone derived from BALB-3T3.. Int J Cancer.

[OCR_00317] Kelsey M. I., Pienta R. J. (1979). Transformation of hamster embryo cells by cholesterol-alpha-epoxide and lithocholic acid.. Cancer Lett.

[OCR_00322] Kennedy A. R., Little J. B. (1978). Protease inhibitors suppress radiation-induced malignant transformation in vitro.. Nature.

[OCR_00327] Kobori O., Shimizu T., Maeda M., Atomi Y., Watanabe J., Shoji M., Morioka Y. (1984). Enhancing effect of bile and bile acid on stomach tumorigenesis induced by N-methyl-N'-nitro-N-nitrosoguanidine in Wistar rats.. J Natl Cancer Inst.

[OCR_00333] Miller K. G., Liu L. F., Englund P. T. (1981). A homogeneous type II DNA topoisomerase from HeLa cell nuclei.. J Biol Chem.

[OCR_00338] Nelson E. M., Tewey K. M., Liu L. F. (1984). Mechanism of antitumor drug action: poisoning of mammalian DNA topoisomerase II on DNA by 4'-(9-acridinylamino)-methanesulfon-m-anisidide.. Proc Natl Acad Sci U S A.

[OCR_00344] Pommier Y., Schwartz R. E., Kohn K. W., Zwelling L. A. (1984). Formation and rejoining of deoxyribonucleic acid double-strand breaks induced in isolated cell nuclei by antineoplastic intercalating agents.. Biochemistry.

[OCR_00350] Reddy B. S. (1981). Dietary fat and its relationship to large bowel cancer.. Cancer Res.

[OCR_00354] Reznikoff C. A., Bertram J. S., Brankow D. W., Heidelberger C. (1973). Quantitative and qualitative studies of chemical transformation of cloned C3H mouse embryo cells sensitive to postconfluence inhibition of cell division.. Cancer Res.

[OCR_00361] Sahyoun N., Wolf M., Besterman J., Hsieh T., Sander M., LeVine H., Chang K. J., Cuatrecasas P. (1986). Protein kinase C phosphorylates topoisomerase II: topoisomerase activation and its possible role in phorbol ester-induced differentiation of HL-60 cells.. Proc Natl Acad Sci U S A.

[OCR_00368] Wang J. C. (1985). DNA topoisomerases.. Annu Rev Biochem.

[OCR_00372] Yang L., Rowe T. C., Nelson E. M., Liu L. F. (1985). In vivo mapping of DNA topoisomerase II-specific cleavage sites on SV40 chromatin.. Cell.

